# Repurposing beta-blockers for combinatory cancer treatment: effects on conventional and immune therapies

**DOI:** 10.3389/fphar.2023.1325050

**Published:** 2024-01-09

**Authors:** Rachel Massalee, Xuefang Cao

**Affiliations:** ^1^ Marlene and Stewart Greenebaum Comprehensive Cancer Center, University of Maryland Baltimore School of Medicine, Baltimore, MD, United States; ^2^ Department of Microbiology and Immunology, University of Maryland Baltimore School of Medicine, Baltimore, MD, United States

**Keywords:** beta blockers, immunotherapy, chemotherapy, selective beta blocker, non selective beta blockers

## Abstract

Beta-adrenergic receptor signaling regulates cellular processes associated with facilitating tumor cell proliferation and dampening anti-tumor immune response. These cellular processes may lead to compromised tumor control and cancer progression. Based on this ramification, Beta-blockers (BBs) have emerged as a potential treatment by inhibiting beta-adrenergic receptor signaling. This review aimed to investigate the relationship between the use of BBs and tumor progression and treatment response. Therefore, the authors explored several aspects: the potential synergistic relationship of BBs with chemotherapy and immunotherapy in enhancing the effectiveness of chemotherapeutic and immunotherapeutic treatments and their role in boosting endogenous immunity. Further, this review explores the distinctions between the major types of BBs: Non-selective Beta Blockers (NSBBs) and Selective Beta Blockers (SBBs), and their contributions to combinatory cancer treatment. In this review, we presented a perspective interpretation of research findings and future directions. Overall, this review discusses the potential and challenge that BBs present in improving the effectiveness and outcome of cancer treatment.

## Introduction

Beta-adrenergic blocking agents, identified as Beta Blockers (BBs), are a family of medications conventionally regarded as cardioprotective. BBs have been well documented for preserving cardiovascular health and reducing mortality rates among individuals diagnosed with hypertension (Argulian et al., 2019). Within the last decade, researchers have studied the potential use of BBs as a therapeutic option for cancer treatment. Naturally, this undertaking has ignited intriguing debates. While some researchers emphasize the advantages of BBs in suppressing cancer growth and amplifying therapeutic responses, as exemplified in a new report (Alaskar et al., 2023), there remains a segment of the scientific community who approach the findings with caution or skepticism due to varying results and the challenges of study methodologies, as shown in a meta-analysis that evaluated BBs on clinical impact in cancer patients (Na et al., 2018), which highlighted the challenges of study methodologies and the caution we must take in interpreting the findings.

Several preclinical studies provided promising results on how BBs impact tumor cells as well as the tumor microenvironment, and also proposed potential molecular and cellular mechanisms (Pasquier et al., 2013; Coelho et al., 2015; Farhoumand et al., 2023). Meanwhile, a number of clinical trials are currently assessing the effects of BBs, focusing on non-selective BBs, on cancer progression and treatment response in combination with chemotherapy or immunotherapy. It became evident that there were differing viewpoints on the mechanisms through which BBs modulate tumor progression and the tumor microenvironment. If we can better understand these pathways, it is possible to combine BBs with conventional and immune therapies to improve treatment outcome for cancer patients. To this end, a comprehensive understanding of the impact of BBs on the tumor immune microenvironment will facilitate the development of novel therapies in the field of immuno-oncology.

The research for this review was performed from July 2023 to November 2023, the authors utilized databases such as Google Scholar, PubMed, and Embase. Several keywords and their integration were used, including “beta-adrenergic signaling,” “tumor microenvironment,” β-blockers,” “effects,” “cancer progression,” and “immunotherapy.” Novel therapeutic approaches can be designed based on improved understanding of the complex interactions among the tumor microenvironment, BBs, and immune response. In hopes of gaining a contextual overview, this review study has attempted to examine the relationship between BBs, chemotherapy, and immunotherapy.

## Improving the effectiveness of cancer treatment: the synergistic potential of beta blockers and chemotherapy

The interplay between BBs and chemotherapy agents in cancer treatment encompasses immense potential. The synergistic potential between BBs and chemotherapy may substantially boost the therapeutic efficacy of chemotherapy treatments by enhancing the antiproliferative effect as well as intensifying its antimitotic and antimitochondrial properties (Pasquier et al., 2013; Coelho et al., 2015; Montoya et al., 2017; Na et al., 2018). The role of microtubule has been well established in various eukaryotic processes from cell division and growth (Singh et al., 2008; Mukhtar et al., 2014). Consequently, microtubule-targeting drugs such as vinblastine or vincristine have been developed to inhibit the microtubule polymerization within cells (Jordan and Kamath, 2007). In a study of murine neuroblastoma model, a combination of BBs with chemotherapy drug such as vincristine was used to heighten the microtubule targeting agent against the neuroblastoma cells which led to a potent inhibition of tumor cell growth (Pasquier et al., 2013). Within this study, the administration of BBs decreased the presence of CD31^+^ cells, which led to the amplification of vincristine effect. Additionally, when examining the effects of BBs, it was observed that a specific beta-blocker, propranolol (PRO), at a toxic concentration, caused fragmentation of the mitochondrial network. Furthermore, the study found that higher levels of toxicity induced by PRO halted the mitotic phase of neuroblastoma cells. Similarly, another study illustrated the possibility of using BBs as a supplementary therapy as it induces cell cycle arrest and promote apoptosis in human colorectal cancer cells (Coelho et al., 2015). As of 2023, a new study demonstrated that the concurrent use of PRO with chemotherapy, exhibits antitumor properties when administered alongside chemotherapy in the context of multiple myeloma cells (Satilmis et al., 2023).

In parallel to those preclinical studies with animal or cell culture models, prolonged overall survival (OS) has been observed in patients diagnosed with epithelial cancer when BBs were used in combination with chemotherapy. The combination treatment in epithelial ovarian cancer patients was associated with longer OS, and was believed to reduce tumor angiogenesis, tumor growth, and cause delays in wound healing (Watkins et al., 2015). A comparable outcome was reported that showed an association between perioperative use of BBs and longer OS in patients undergoing primary ovarian cancer cytoreductive surgery (Al-Niaimi et al., 2016). Several other studies have demonstrated a consistent increase in OS with BBs in combination with selected therapeutic approaches in breast and esophageal cancer patients (Farrugia et al., 2020; Løfling et al., 2022) where the perioperative use of BBs alongside chemoradiation treatment also extended OS. Additionally, BB usage was reported to contribute to improved survival for breast cancer patients (Raimondi et al., 2016). As a promising observation of the endogenous immune response, the combination treatment with BBs and chemotherapy increases cellular immunity by enhancing NK cell function (Chung et al., 2016). Another retrospective study investigated utilizing BBs alongside chemoradiotherapy for unresectable stage III non-small-cell lung cancer. The study highlighted cardiovascular complications such as cardiac arrhythmia and cardiac ischemia that could emerge during chemoradiotherapy. BBs were found to play a crucial cardioprotective role by mitigating the damage to the heart. Moreover, the combination of BBs with chemoradiotherapy could lead to longer OS. The dual action of BBs seemed to shield the heart from the adverse effects of cancer treatments but also improve endogenous tumor immunity, as evidenced by enhanced presence of CD8^+^ cytotoxic T lymphocytes (Zaborowska-Szmit et al., 2023).

These studies suggest that combining BBs with chemotherapy creates synergistic effects, resulting in better tumor control, making it difficult for the tumors to evade the treatments. At the cellular and molecular level, the combination of BBs and chemotherapy is expected to create an anti-proliferative effect as well as intensify antimitotic and antimitochondrial properties. This synergism improves patient outcomes as the combination treatment leads to better tumor regression. These results and concepts provide a strong rationale for prospective clinical trials that test the combination of BBs and chemotherapy for various types of cancer patients.

## Enhancing cancer immunotherapy with beta blockers

Manipulating the beta-adrenergic signaling pathways as potential targets for cancer treatment requires a deep comprehension of their mechanisms. The significance of the immune system in cancer development and treatment response is well established. Prior studies have shown that β2-AR signaling in immune cells regulate immune response in cancer and inflammation models (Wang and Cao, 2019; Thapa and Cao, 2023). For instance, β2-ARs expressed on CD8^+^ T cells hinder their migratory and cytotoxic activities, indicating an immunosuppressive effect within the tumor immune microenvironment (Mravec et al., 2020). In addition, β2-AR-stimulated suppression of T cells and antigen-presenting cells has been demonstrated in allogeneic hematopoietic cell transplantation models (Leigh et al., 2015; Mohammadpour et al., 2018; Mohammadpour et al., 2020). These immunosuppressive effects suggest that β-adrenergic signaling acts as an immune checkpoint during tumor development or progression. Therefore, inhibiting β2-AR signaling may have the potential to enhance anti-tumor immune response, and the combination of BBs with immunotherapy may further improve cancer treatment.

**FIGURE 1:**
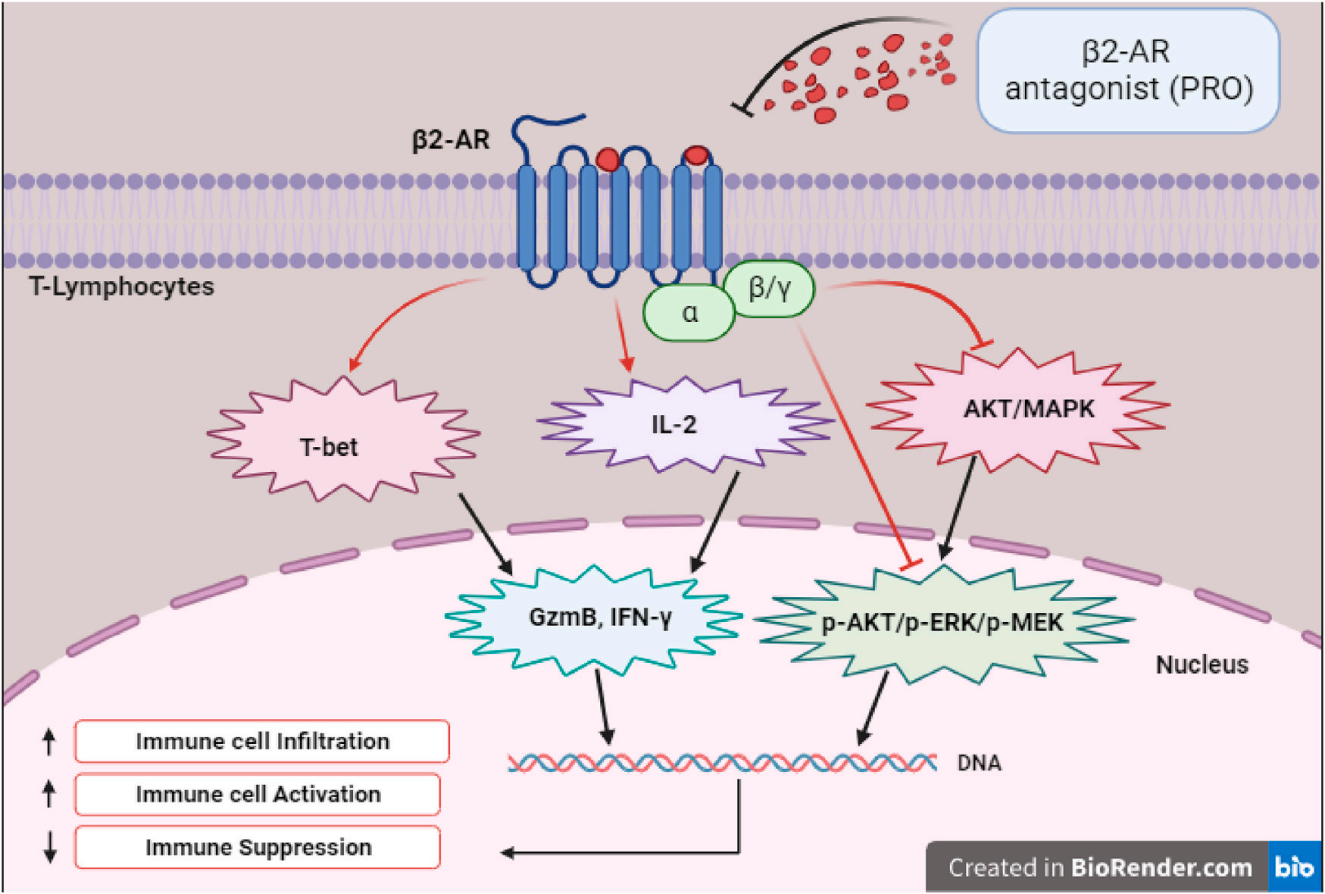
Depicts the transcription factors, signaling, and effector molecules involved in the upregulation of immune response following β2-AR blockade.

As illustrated in Figure 1, experiments with specific BBs like PRO demonstrated that the inhibition of β2-AR enhances the functionality of CD8^+^ T cells, which leads to improved tumor control. Studies with mouse models of colon and breast cancers have established that blocking β2-AR enhanced anti-tumor immunity (Farooq et al., 2023). Moreover, it has been demonstrated that β2-AR antagonist like PRO not only regulates p-AKT/p-ERK/p-MEK and AKT/MAPK pathways but also increases T-bet signaling to enhance the expression of GzmB and IFN-γ effector molecules. Clinical studies investigating the impact of BBs on lung cancer patients undergoing immune checkpoint blockade therapy revealed that the use of BBs enhanced overall progression free survival (Oh et al., 2021). Furthermore, BBs were associated with better OS in a study that investigated the effects of wide spectrum BBs, particularly PRO, on melanoma progression in both animal models and human patients. PRO was found to increase the infiltration of T-lymphocytes and cytotoxic activity in tumors, while tumor cells and macrophages may generate increased beta-adrenergic signaling in the tumor microenvironment (Wrobel et al., 2020). Though this study did not have immunotherapy as a treatment, they suggested that BBs may affect the immune profiles and melanoma progression. Another study observed that BBs, when combined with immunotherapy, prolonged OS in mice by enhancing immune cell activities, specifically enhancing the responsiveness of immune cells to IL-2 (Kokolus et al., 2018).

To understand the specific cellular mechanisms of how BBs work in conjunction with immunotherapy, recent research in 2023 indicated that PRO combined with Immunotherapy, such as program death ligand 1 (PD-L1) inhibitors could prevent PD-L1 overexpression in ovarian cancer. This proposes a potentially more targeted response to PD-L1 inhibitors due to possible intrinsic PD-L1 expression in cancer cells (Falcinelli et al., 2023). The implication of this finding suggests that BBs could enhance the effectiveness of immune checkpoint therapy. Prior to the study by Falcinelli et al., a meta-analysis and retrospective study published in 2022 aimed to explore the impact of BBs on various types of checkpoint inhibitors (ICI) such as PD-1 inhibitors (Yan et al., 2022). It was discovered that BB use may be associated with improved anticancer efficacy of ICIs. Additionally, a recent clinical trial investigated the synergy between immunotherapy and BBs. The study revealed that pembrolizumab, a PD-1 blocking monoclonal antibody, exhibited significant recurrence-free survival for melanoma patients when combined with BBs in comparison to placebo, indicating a potential synergetic effect between immune checkpoint blockade and BBs. These studies suggest that the combination of immunotherapy with BBs represents a promising avenue for further investigation in melanoma treatment (Kennedy and Neary, 2022). In-depth research is necessary to comprehensively unravel the cellular mechanisms underlying how BBs and immunotherapy contribute to creating an antitumor microenvironment.

## Non-selective beta blockers vs. selective beta blockers (NSBB vs. SBB) in cancer treatment

It is important to recognize that the effectiveness of the combination treatment is influenced by two types of BBs. BBs are either classified as non-selective beta blockers (NSBB) or selective beta blockers (SBB) (Table 1). The NSBBs bind to the β1-and β2-receptors and inhibit both receptors; an example of NSBB would be propranolol (PRO) and carvedilol. On the other hand, β1-SBBs have a β1 selectivity causing an antagonizing effect on the β1-receptor; examples of β1-SBB include isoprolol, metoprolol, and acebutolol (Ripley and Saseen, 2014; Farzam and Jan, 2022). Likewise, β2-SBBs selectively target the β2-receptor; Examples of β2-SBBs include ICI and Butoxamine. Recognizing the distinction is vital for understanding their effectiveness.

**TABLE 1:** Comparison of Non-selective Beta Blocker (NSBB) vs. Selective Beta Blocker (SBB).

*Types of Beta Blockers (BBs)*	*Examples of BBs*	*Mechanisms of Action*	*Impact on tumor proliferation*	*Impact on overall survival (OS)*
Non-selective beta blocker (NSBB)	Propranolol	Inhibition of β1-and β2-receptors	Significant decrease in tumor proliferation (Montoya et al., 2017)	A loner OS in patients. Example patients with breast cancer and epithelial ovarian cancer (Watkins et al., 2015; Montoya et al., 2017)
	Carvedilol			
	Nadolol			
	Labetalol			
	Levobunolol			
	Sotalol			
	Timolol			
	Oxprenolol			
Selective Beta1 blocker (SBB)	Isoprolol	Selective inhibition of β1- receptor	Impact varies: Not as potent as NSBB in tumor cell proliferation (Montoya et at., 2017).	Impact varies on the specific type of SBB to impact OS (Farhoumand et al., 2023).
	Metoprolol			
	Acebutolol			
	Atenolol			
	Betaxolol			
	Celiprolol			
	Esmolol			
Selective Beta2 blocker (SBB)	ICI	Selective inhibition of β2-receptor	Comparable effects to NSBB if β2 inhibition (ICI) occurs (Cuesta et al., 2019; Del Vecchio et al., 2023; Satilmis et al., 2023)	Comparable to NSBB if β2 inhibition (ICI) occurs (Cuesta et al., 2019; Del Vecchio et al., 2023; Satilmis et al., 2023)
	Butoxamine			

**FIGURE 2:**
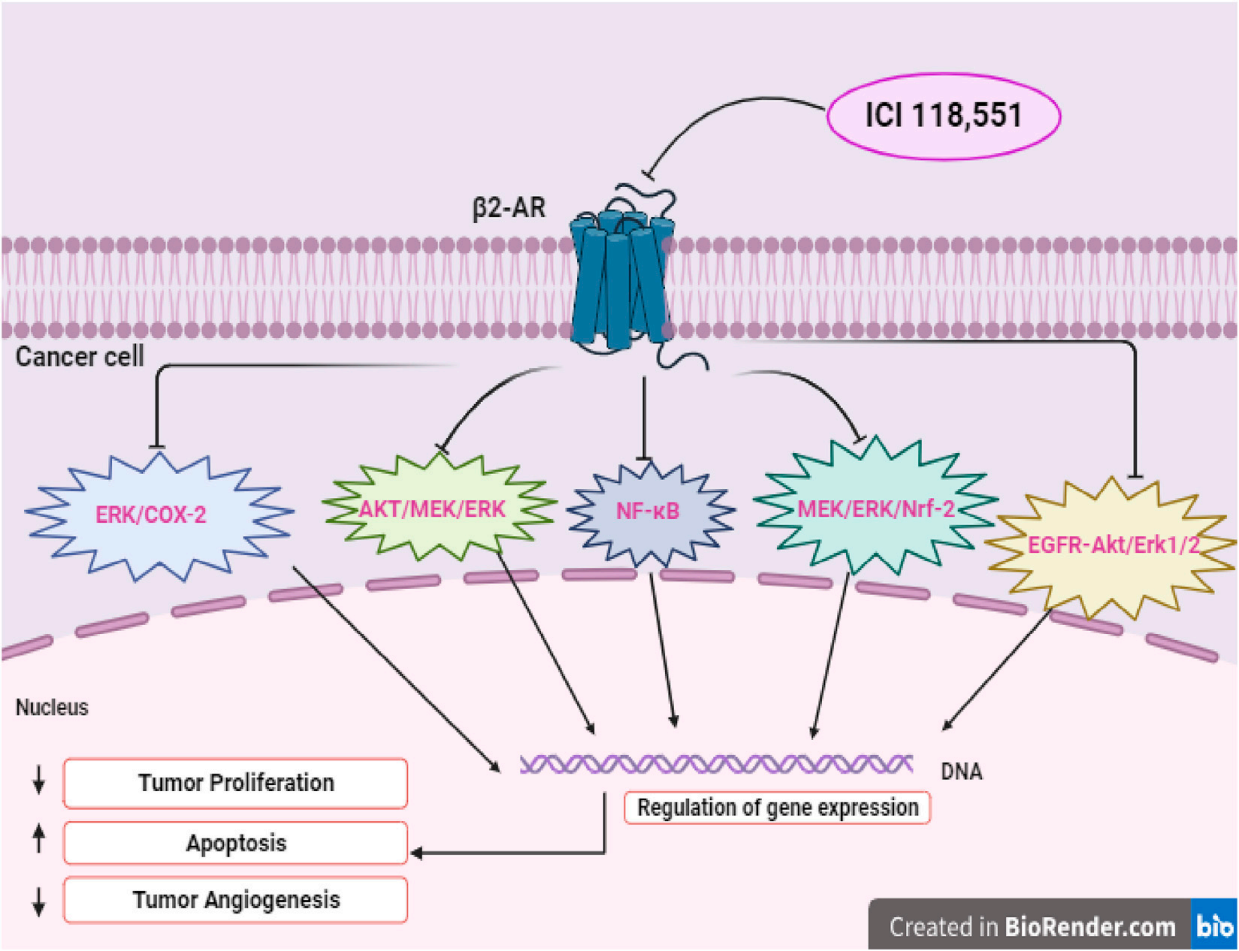
Illustrates the potential signaling pathways of selective β2-blocker, ICI-118,551 (ICI), showcasing direct effect on tumor cells.

Several studies have explored the outcomes of cancer progression and OS with NSBB and SBB. A retrospective study was carried out within a population of patients diagnosed with breast cancer to understand the association between BBs and the proliferation of breast cancer. It was found that NSBB, compared to SBB, showed significant decrease in tumor proliferation. To validate their finding, the researchers conducted a prospective study where breast cancer patients were given NSBB, PRO. The results indicate a significant decrease in tumor proliferation (Montoya et al., 2017). Another study examined individual patients diagnosed with epithelial ovarian cancer and found that patients who received the NSBB had longer OS compared to the patients assigned to the SBB group (Watkins et al., 2015). Interestingly, recent literature suggested that if an SBB inhibits the β2-receptor, its effect is comparable to that of an NSBB. Studies have indicated that a specific selective β2-blocker, ICI-118,551 (ICI) induces apoptosis in cancer cells. In a study looking at hemangioblastomas, the use of ICI was found to block the β2-receptor, impairing the viability of cancer cells by inducing apoptosis. It was found that ICI mirrored the previous results obtained with NSBB, PRO, which inhibits both the β1-and β2-receptors (Cuesta et al., 2019). In the subsequent study, the anti-tumor effects of ICI within a pre-clinical *in vivo* model were investigated in head and neck cancer. It was revealed that ICI in combination with Cetuximab (CTX) treatment resulted in a significant decrease in tumor growth due to several factors: the inhibition of the ERK phosphorylation as well as an impairment within the MEK/ERK/Nrf-2 axis pathway (Del Vecchio et al., 2023). Figure 2 illustrates a possible schematic representation of the ICI on the β2-AR as it leads to the downregulation and inhibition of several pathways such as MEK/ERK/Nrf-2,EGFR-Akt/Erk1/2,ERK/COX-2,AKT/MEK/ERK, and NF-κB. Downregulation of these pathways induces apoptosis, cell cycle arrest at G0/G1 and S Phase, and overall suppression of various cellular pathways (Shan et al., 2011; Xie et al., 2019; Li et al., 2022; Del Vecchio et al., 2023; Shi et al., 2023). Furthermore, another study aimed to assess the antitumor effects of NSBB and SBB in multiple myeloma when combined with chemotherapy. It was concluded that BBs that targets β2-AR from PRO to ICI affected multiple myeloma cell viability and induced apoptosis and autophagy (Satilmis et al., 2023).

These findings suggest that antagonistic effects on β2-AR might be more potent in exacerbating tumor cell death. The studies reveal that NSBB and SBB target the beta-receptors in various ways which lead to different impacts on treatment outcome for cancer patients. Though they both impact cancer progression, it is essential to acknowledge that NSBB in comparison to SBB demonstrated a significant reduction in tumor proliferation and improved OS with patients. Notably, the specific selective β2-receptor blocker, ICI, demonstrated effects similar to the NSBB. Additional research is required to understand the mechanisms and explore the translational possibilities.

## Concluding remarks and future directions

We have explored the relationship between BBs and cancer progression in the setting of conventional and immune therapies. Beginning with the role of beta-adrenergic signaling pathway in fostering malignant progression, we are encouraged by the potential of using BBs to inhibit the adrenergic pathway for the benefit of cancer treatment. We also delved into the synergistic relationship of BBs with immunotherapy that unveiled a promising avenue for cancer treatment by using BBs to enhance the functionality of immune cells in particular CD8^+^ T cells leading to better tumor control. We also view that the combination of an NSBB with immunotherapy can counteract tumor-induced immune suppression thereby improving the efficacy of immunotherapy.

Clinical trials are critical in developing and implementing novel treatments. A clinical trial under the identifier NCT02013492 used NSBB to treat patients with metastatic tumors to discern the effects of BBs on the tumor microenvironment and the host immune system. Additionally, a completed trial, NCT01847001, utilized NSBB for breast cancer patients undergoing chemotherapy. An upcoming study, NCT05968690, will investigate BBs as an added treatment alongside immunotherapy. These studies, among many others, punctuate the importance of exploring the role of BBs in combination with chemotherapy and immunotherapy for cancer treatment.

It would be remiss not to acknowledge articles that question the beneficial effects of BBs as adjunctive treatments, particularly in combination with immunotherapy. A recent study investigated the benefits of utilizing BBs in hepatocellular carcinoma (HCC) patients treated with ICIs. The researchers concluded that BB use was not associated with improved OS, PFS, or ORR for unresectable HCC patients treated with immunotherapy (Wu et al., 2023). While we acknowledge the possibility of using BBs as a supplemental cancer treatment, a comprehensive understanding of the literature will assist in addressing challenges and limitations that might arise. It is important to be cognizant that variations within cancer types could impact the effectiveness of BBs on immunotherapy or chemotherapy.

In summary, this review delved into the intricate relationships between BBs and cancer treatment including chemotherapy and immunotherapy and the potential amplifier effects on such therapies. The use of BBs in a combination treatment is significant in modulating the tumor microenvironment and boosting immune response. We explored molecular mechanisms and the relevant pathways. However, further studies are necessary in both the clinical setting and animal models to fully comprehend the molecular mechanisms and validate the potential synergistic relationship between BBs, chemotherapy, and immunotherapy. Continuous research on this topic is vital for improving treatment outcome and quality of life for cancer patients.
